# Medical Nutrition Therapy in Critically Ill Patients with COVID-19—A Single-Center Observational Study

**DOI:** 10.3390/nu15051086

**Published:** 2023-02-22

**Authors:** Łukasz J. Krzych, Maria Taborek, Katarzyna Winiarska, Justyna Danel, Agnieszka Nowotarska, Tomasz Jaworski

**Affiliations:** 1 Department of Anaesthesiology and Intensive Care, Faculty of Medical Sciences in Katowice, Medical University of Silesia, 14 Medyków Street, 40-752 Katowice, Poland; 2 Students’ Scientific Society, Department of Anaesthesiology and Intensive Care, Faculty of Medical Sciences in Katowice, Medical University of Silesia, 40-752 Katowice, Poland

**Keywords:** COVID-19, SARS-CoV-2, nutrition support, medical nutrition therapy, mechanical ventilation, prone position, intensive care

## Abstract

Medical nutrition should be tailored to cover a patient’s needs, taking into account medical and organizational possibilities and obstacles. This observational study aimed to assess calories and protein delivery in critically ill patients with COVID-19. The study group comprised 72 subjects hospitalized in the intensive care unit (ICU) during the second and third SARS-CoV-2 waves in Poland. The caloric demand was calculated using the Harris–Benedict equation (HB), the Mifflin–St Jeor equation (MsJ), and the formula recommended by the European Society for Clinical Nutrition and Metabolism (ESPEN). Protein demand was calculated using ESPEN guidelines. Total daily calorie and protein intakes were collected during the first week of the ICU stay. The median coverages of the basal metabolic rate (BMR) during day 4 and day 7 of the ICU stay reached: 72% and 69% (HB), 74% and 76% (MsJ), and 73% and 71% (ESPEN), respectively. The median fulfillment of recommended protein intake was 40% on day 4 and 43% on day 7. The type of respiratory support influenced nutrition delivery. A need for ventilation in the prone position was the main difficulty to guarantee proper nutritional support. Systemic organizational improvement is needed to fulfill nutritional recommendations in this clinical scenario.

## 1. Introduction

The severity of acute respiratory distress syndrome (ARDS) is the key factor determining a need for respiratory support during COVID-19. Three subphenotypes of COVID-19 were distinguished. They vary in expression of inflammatory cytokines, which determine the acuity of respiratory failure, preferred treatment strategy, and outcome of patients [1]. Even one in ten SARS-CoV-2 infected patients is at risk of severe manifestation of the disease and may require admission to the intensive care unit (ICU) due to acute respiratory failure or multiorgan dysfunction [2], and severe dysregulation of the immune system in subphenotype-1 COVID-19 group is associated with the highest mortality [1]. Respiratory support may vary from oxygen therapy delivered by nasal cannula or mask, via different modes of mechanical support, preferably reflecting the concept of safe mechanical ventilation, to extracorporeal membrane oxygenation [3,4].

Critical illness with cytokine storm and hyperinflammation is associated with catabolism, leading to significant weight reduction and severe loss of skeletal muscles [5]. Decreased intake of nutrition products and increased digestive losses associated with the course of the disease are additional reasons for malnutrition [6], which worsens prognosis [7]. The experts’ statement of the European Society for Clinical Nutrition and Metabolism (ESPEN) and practical guidelines for the nutritional management of individuals with SARS-CoV-2 infection [5] and ESPEN guidelines on clinical nutrition in the ICU [8] underline the significance of proper medical nutrition therapy, which has become an integral part of modern critical care.

In this study, we aimed to assess clinical practice in terms of delivering calorie and protein support in COVID-19 critically ill patients with respiratory failure who required invasive mechanical ventilation due to severe ARDS.

## 2. Materials and Methods

We performed an observational study covering COVID-19 patients hospitalized during the second and third SARS-CoV-2 waves in Poland (from Oct 2020 to May 2021). We screened consecutive critically ill adults treated in the mixed intensive care unit (ICU) at a university hospital in Katowice, Poland. None of the patients had significant clinical contraindications to be included into this observation (i.e., burns; chronic, preexisting neuromuscular, psychiatric, or neurological conditions with cachexia; home nutritional support or chronic mechanical ventilation before or at the time of ICU admission; palliative or end-of-life care). Due to the non-interventional nature of the study, informed consent was not required from patients to participate. To avoid potential influence on the results, the ICU treatment team had no knowledge of the planned data analysis.

Demographic data, the severity of critical illness using the Acute Physiology and Chronic Health Evaluation II (APACHE) score, the Simplified Acute Physiology Score (SAPS) II, and the Sequential Organ Failure Assessment (SOFA) score. The values were calculated using the online MDCalc calculator (ww.mdcalc.com). Nutritional status using the Nutritional Risk Screening tool (NRS) and frailty using the Clinical Frailty Scale (CFS) were assessed at the bedside on admission as well. On the day of admission to the intensive care unit and throughout ICU stay, patients were monitored in accordance with best clinical practice and underwent a full assessment of biochemical parameters (incl. creatinine, blood urea nitrogen, bilirubin, alkaline phosphatase, aspartate aminotransferase, alanine aminotransferase, gamma-glutamyl transferase, lactate dehydrogenase, albumins, Na^+^, K^+^, Cl^−^). Patient-centered monitoring was applied in all cases. Extensive assessment of vital signs was applied, including monitoring of hourly diuresis, body temperature, invasive blood pressure, heart rate via ECG, respiratory rate, and type and parameters of mechanical ventilation. If indicated, advanced hemodynamic monitoring using APCO or PICCO was used. Clinical variables were recorded from admission until ICU discharge, death, or day 7 of the ICU stay. Furthermore, data on the length of hospitalization before ICU admission were collected. 

All patients received tailored medical nutrition therapy, based on their current clinical status. The route, type, and dose of the prescribed nutrition were left to the discretion of the treating physician. To calculate the energy demand, i.e., to define the basal metabolic rate (BMR), the following formulas were used: Harris–Benedict equation (HB), Mifflin–St, Jeor equation (MsJ), and the ESPEN formula (i.e., 20 kcal/kg). The first two equations use mathematical formulas to determine BMR. Both use the following data: weight (W) in kilograms, height (H) in centimeters, and, in the case of MsJ, age, and vary according to the gender of the patient being assessed. For the Mifflin–St Jeor Equation, we used for men: BMR = 10 W + 6.25 H − 5 A + 5, and for women: BMR = 10 W + 6.25 H − 5 A − 16 [9]. In the case of the revised Harris–Benedict Equation for men: BMR = 13.397 W + 4.799 H – 5.677 A + 88.362 and for women BMR = 9.247 W + 3.098 H − 4.330 A + 447.593 was used [10]. Calories intakes were calculated from all nutritional sources, i.e., enteral nutrition (EN), parenteral nutrition (PN), supplemental parenteral nutrition (SPN), and oral nutrition (ON), including oral nutritional supplements (ONS), non-nutritional calories, including propofol, citrate, or glucose. Calculations were based on the patient’s actual body weight measured on admission. Indirect calorimetry was inaccessible during the observation period. Calorie and protein intake was assessed on a daily basis till day 7 of the ICU stay or patient’s death, whatever occurred first. The assessment of the calories and protein intake amount was carried out by researchers. Data was collected on the basis of daily nursing reports containing: type of nutrition (EN, PN, OR, MIX), trade name of nutritional preparation, flow of nutritional specimens (if applicable), period of nutrition administration. Detailed information about nutritional value (calories and protein) was obtained from information provided on pharmaceutical labels. All possible breaks in the use of nutritional supplements (e.g., while prone) and changes in nutritional supplements were taken into account. The fulfillment of recommendations in terms of delivered calories and proteins was calculated for seven consecutive days. Provision of nutrition was assessed in terms of illness acuity on admission, type of respiratory support (oxygen therapy with face mask or nasal cannula, high low nasal oxygen therapy, non-invasive ventilation or continuous positive airway pressure support, invasive mechanical ventilation) and position in which the patient was ventilated (supine or prone).

Statistical analysis was performed using MedCalc v.18 software (MedCalc Software, Ostend, Belgium). Quantitative variables were shown using medians and interquartile ranges (IQR). Qualitative data were expressed using frequencies and percentages. Correlations were assessed by Spearman’s rank correlation coefficient. The Mann–Whitney or Student’s t-test was applied to verify the between-group differences for independent continuous variables. The Wilcoxon signed-rank test for paired samples was used for dependent data. The chi-squared or the Fisher exact test was applied to verify the differences for qualitative variables. All tests were two-sided. A *p*-value of <0.05 was considered significant.

## 3. Results

The study group comprised 72 subjects, aged 37–83 (median 63, IQR 58–70) years. Patients included in the study spent a median of 5.5 days (2–14) in other hospital wards due to SARS-CoV-2 infection. The characteristics of the subjects on admission are shown in Table 1. On day 7, 51 (71%) persons were still hospitalized in the ICU. Mortality during the overall ICU stay reached 65%.

Regarding respiratory support on ICU admission, 5 (7%) patients required oxygen therapy with face mask or nasal cannula (OX), 3 (4%) had high flow nasal oxygen therapy (HFNOT), 15 (21%) had non-invasive ventilation or continuous positive airway pressure support (NIV), and 48 (67%) required invasive mechanical ventilation (IMV). Progression of the ARDS with a need for any escalation of respiratory therapy was observed in all remaining patients (n = 23, 32%). Taken altogether, all patients were mechanically ventilated for at least 1 day during our observation period. None of the patients either received or qualified for extracorporeal membrane oxygenation.

The median number of days of OX was 3 (IQR 1–4), for HFNOT it was 1 (IQR 1–2), for NIV it was 4 (IQR 2–6), and for IMV it was 4 (IQR 2–7). Ventilation in a prone position was applied in all subjects, including those requiring IMV. Conscious patients were advised to change position in bed as many times as possible daily, promoting recruitment of the affected lung, and it was impossible to calculate the total number of hours in each position. Prone positioning was achievable in 38 (53%) of mechanically ventilated patients and it lasted for a median of 40 (IQR 24–55.5) hours per patient per stay. All patients required a reduction in the quantity and 35 (92%) required even termination of the planned dose of EN in a prone position due to regurgitations. All patients receiving prone positioning had muscle relaxants.

A total of 62 (86%) patients required hemodynamic support with catecholamines for at least one day with a median duration of 4 (3–6) days. A total of 11 (15%) subjects had continuous renal replacement therapy with a median duration of 4 (IQR 2–5) days. All patients received regional anticoagulation with citrate.

Patients’ nutritional status was altered with the length of hospitalization before ICU admission. There was a statistically significant positive correlation between the length of hospitalization and the NRS (r = 0.37, *p* < 0.05). Nutrition in ICU was commenced on the first day of stay among 56 (78%) of patients. Statistically, EN was started on day 2 (IQR 2–4), PN on day 1 (IQR 1–3), and ON was commenced on day 4 (IQR 4–6). Non-nutritional calories were delivered to all subjects. The median duration of EN was 5.0 (4.0–6.0) days, 1.0 (1.0–3.0) days for PN, and 2.0 (2.0–3.0) days for SPN.

Table 2 demonstrates the delivery of energy. Table 3 presents protein intake. The provision of calories and proteins increased progressively over the first 4 days. On day 4, patients met on average 74 (36–118)% of BMR targets and 40 (19–61)% of the ESPEN COVID-19 protein targets. On day 7, patients met on average 76 (52–101)% of BMR targets and 43 (29–65)% of the ESPEN COVID-19 protein targets. Patients who received SPN were most often overfed.

**Figure 1:**
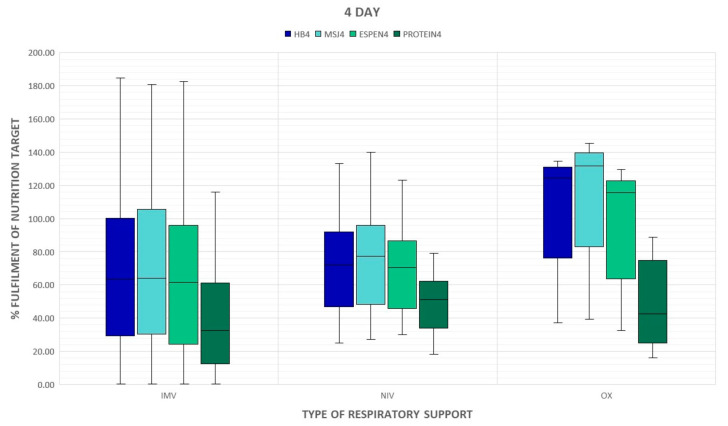
Type of respiratory support at the ICU admission and delivery of energy and proteins on day 4. BH: Harris–Benedict equation; MsJ: Mifflin–St. Jeor equation; OX: passive oxygen therapy; NIV: non-invasive ventilation; IMV: invasive mechanical ventilation.

**Figure 2:**
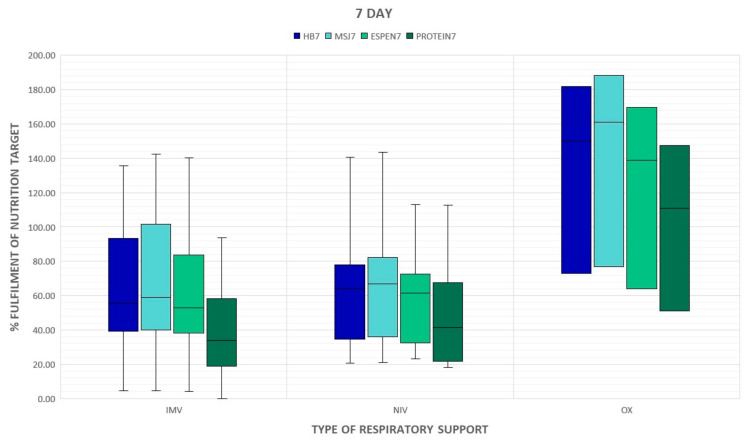
Type of respiratory support at the ICU admission and delivery of energy and proteins on day 7. BH: Harris–Benedict equation; MsJ: Mifflin–St. Jeor equation; OX: passive oxygen therapy; NIV: non-invasive ventilation; IMV: invasive mechanical ventilation.

**Figure 3:**
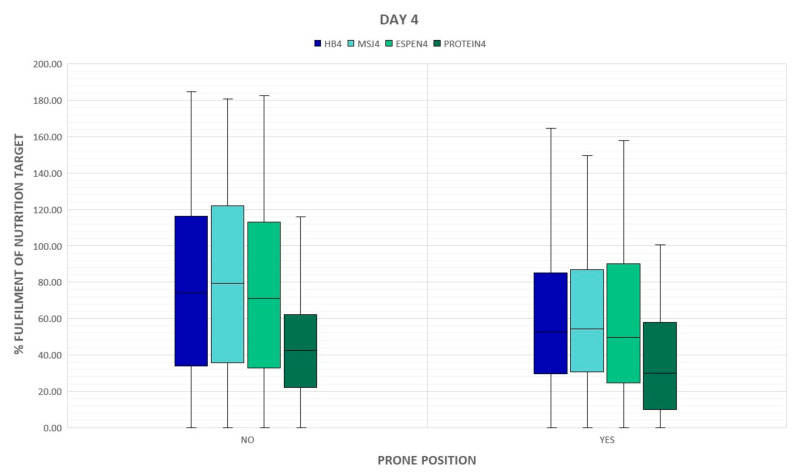
Prone positioning during ICU stay and delivery of energy and proteins on day 4. BH: Harris–Benedict equation; MsJ: Mifflin–St. Jeor equation; OX: passive oxygen therapy; NIV: non-invasive ventilation; IMV: invasive mechanical ventilation.

**Figure 4:**
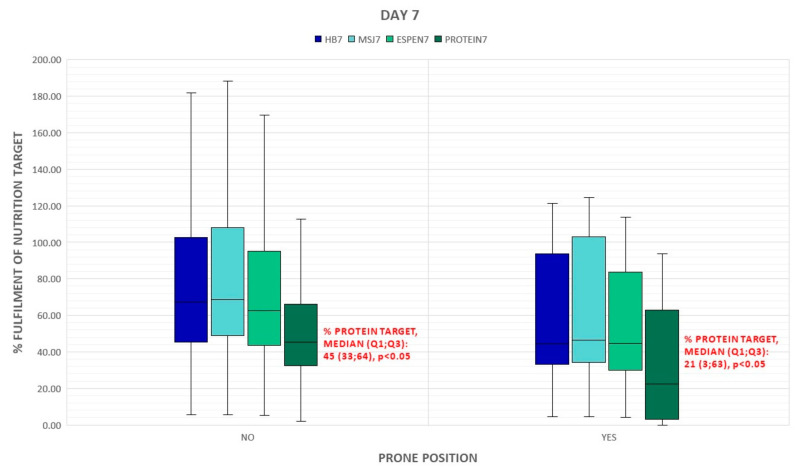
Prone positioning during ICU stay and delivery of energy and proteins on day 7. BH: Harris–Benedict equation; MsJ: Mifflin–St, Jeor equation; OX: passive oxygen therapy; NIV: non-invasive ventilation; IMV: invasive mechanical ventilation.

The percent of energy and protein delivered on day 7 statistically significantly correlated with the acuity of illness assessed by SAPS II on admission but this association was poor (Table 4). The type of respiratory support did not have a significant impact on the supply of calories and proteins, however, a downward trend in the provision of calories and protein in mechanically ventilated groups is noticed (Figure 1 and Figure 2). Patients who required prone positioning (regardless of the type of respiratory support) received less energy and proteins (Figure 3 and Figure 4).

## 4. Discussion

Considering multiple factors, such as older age, comorbidity, prolonged stay in the ICU, reduced oral intake, changes in mobility, increased catabolism, and inflammation leading to malnutrition in COVID-19 patients [5], proper nutritional intervention is an important part of treatment. In this study, the caloric goals estimated using the Harris–Benedict equation, the Mifflin–St. Jeor equation, and the ESPEN recommendation (20 kcal/kg) [8] for each day were not achieved and approximately half of the analyzed group received hypocaloric nutrition until the seventh day of stay in the ICU, when the ESPEN protein target was implemented only in 43%. Neither hypocaloric nutrition nor protein intake had an impact on the outcome in the observed group. Decreased calories and protein supply on the 7th day of observation weakly correlated with higher SAPS II score on admission in regards to ESPEN formulas. The primary acuity of the disease does not predict the approach to nutritional treatment. ESPEN guidelines [8] recommend early nutritional support with progressively increased calorie and protein intake. Patients in shock, including those who require vasopressors, who usually present with multiorgan failure and achieve higher scores in the preliminary assessment of the severity of the illness, should also receive nutrition therapy after their condition stabilizes. This may result in a delay in nutrition, however, in the present study nutritional therapy was implemented early in the therapeutic process, on average on the 1st day of ICU stay.

The type of ventilation route implies restrictions on how the patient is nourished. In the study group, 21% of the patients required NIV and 67% were mechanically ventilated on admission. Inadequate protein intake is common in both mentioned types of respiratory support [11]. During noninvasive ventilation, occurring respiratory complications such as air leakage or altered diaphragmatic function may lead to late implementation of EN feeding, which can affect the nutritional status of the patients [5,12]. As the ARDS progressed, the patient’s respiratory effort began to be insufficient to provide adequate gas exchange in the lungs, and in 32% of the study group IMV was necessary. In the presented study, patients were mechanically ventilated for a median of 4 days during the observation period, which could preclude providing energy and protein demands.

Negative energy balance used to be related to acute respiratory distress syndrome, prolonged mechanical ventilation [13], sepsis, renal failure, increased total complication rate [14], and prolonged ICU stay [13]. Although meta-analyses are in contradiction to the studies mentioned above, presenting no differences in mortality, mechanical ventilation period, duration of stay in ICU, general hospitalization period, and acquired infections between the group receiving a hypocaloric and isocaloric diet [15,16,17], malnutrition is certainly deleterious, even if accurate energy target in the ICU is still unknown. Observational studies indicate that higher protein intake is associated with improved survival [13,18,19,20]. If the caloric intake among ICU patients is still a matter of debate, the importance of protein supply is greatly acknowledged. A recent study “Medical nutrition therapy and clinical outcomes in critically ill adults: A European multinational, prospective observational cohort study (EuroPN)”, demonstrated that patients receiving moderate calories intake defined as 10–20 kcal/kg [21] had longer survival time and shorter mechanical ventilation period compared to a lower calorie intake (<10 kcal/kg), whereas higher calorie intake (>20 kcal/kg) was not associated with better prognosis. The study mentioned above also demonstrated low protein intake, with implementation, on average 65% of ESPEN protein targets [21], suggesting that deficits in protein supply are a common concern among ICUs in Europe. In order to provide protein targets, the use of high protein formulas or additional protein supplementation should be considered a standard practice, since the composition of regular nutritional formulas causes difficulties with proper protein administration without refeeding or overfeeding [8,22,23]. Calorie intake above 110% of target has a detrimental impact on clinical condition and is related to increased insulin demand and gastrointestinal intolerance during enteral nutrition [24,25].

The current ESPEN guidelines [8] on clinical nutrition in the ICU support enteral nutrition rather than parenteral nutrition, even among prone patients with ARDS. In a multi-center, retrospective study, Langer et. al. confirmed that the prone position should be intensified in the course of ARDS due to COVID-19 [26]. The duration of prone positioning is positively correlated with clinical outcomes [27,28]. In the presented study, prone position was used in more than half of the patients (53%) and was maintained for an average of 40 h per patient. Although it is recommended to maintain enteral nutrition during pronation [5], we observed a downward trend in calories and protein supply among patients in the prone position. This trend occurred regardless of the day of assessment, and the patients pronated on the 7th day received significantly less protein than the patients who were in a supine position (*p* = 0.025). Efforts made to continue the prone position directly affected the achievement of nutritional goals. Gastric complications during prone positioning, including regurgitations, vomiting, and a high gastric residual volume, can lead to decreased doses of nutrition formula or the switch to parenteral nutrition and can result in poorer compliance with nutritional recommendations in patients undergoing abdominal rotation. Implementing nutritional protocols supports the improvement of nutrition therapy, especially among patients with prone positioning [29,30].

Considering the multiplicity of variables affecting the condition of critically ill patients, which is influenced by the nutritional interventions, further research is needed. In future studies, the direct role of nutritional therapy in the treatment of patients in ICU receiving ventilatory support should be considered, particularly in the terms of tailored nutritional treatment and its impact on patient outcomes.

The limitations of this study should be mentioned. Firstly, the retrospective nature of this study involves the risk of bias. All data, including anthropometric parameters, used to measure energy expenditure were acquired from medical records. It should be emphasized, that the patients analyzed in the presented study constitute a specific group of patients, in whom an additional physical barrier between the medical staff and the patient could have affected the quality of medical records. The unavailability of calorimetry is another limitation. Among ICU patients the use of mathematical formulas (including BMR equations and the ESPEN formula) may result in differences between estimated energy targets and actual patient demand. However, the use of calorimetry would affect the differences in nutritional interventions depending on the method of ventilation, contributing to the incomparability of the results. Thirdly, we did not assess the extension of cytokine storm, which is associated with COVID-19 severity and the risk of hypercatabolism. Of note, the nutritional status of the patients depends on multiple variables. Any infection impairs nutritional status and causes metabolic changes. Although this study contains data about the length of hospitalization previous to ICU admission, we cannot access the total duration and severity of SARS-CoV-2 infection, e.g., the day of positive test results. Finally, a larger sample size could ensure more sufficient power of analysis. The number of included patients was limited, as the study was single-centered and considered only patients with COVID-19. Due to the structure of the study, data comparison with other centers was impossible, which should be taken into account in the future.

## 5. Conclusions

The nutritional treatment of critically ill patients in ICU is essential as well as challenging. Difficulties with accomplishing all recommended targets appeared among patients with COVID-19. Patients’ initial condition, length of hospitalization, type of nutritional intervention, prone position, and type of respiratory support may disturb both energy and protein intake. Therefore, there is a need for improving the strategy of nutritional intervention in this clinical scenario.

## Figures and Tables

**Table 1:** Characteristics of the study population (at ICU admission).

Category	No. (%) or Median (Q1–Q3)
Males/Females	47 (65%)/25 (35%)
BMI [kg m^−2^]	29 (25–34)
APACHE II [points]	18 (14–25)
SAPS II [points]	38.5 (30–49.5)
NRS score [points]	4 (3–5)
CFS score [points]	4 (3–5)
Albumin concentration [g dL^−1^]	2.8 (2.5–3.0)
C-Reactive Protein concentration [mg dL^−1^]	101 (48,1–150)
D-dimer concentration [ng mL^−1^]	2739 (1449–6952.5)
Lactate dehydrogenase level [U L^−1^]	559 (352–715.15)
Total lymphocyte count [10^3^ µL^−1^]	0.6 (0.39–0.89)
SOFA score [points]	9 (5–12)
Acute respiratory failure with oxygen support	74 (100%)
Oxygenation Index < 100 [mmHg]	11 (15%)
Oxygenation Index 100–300 [mmHg]	57 (79%)
Oxygenation Index > 300 [mmHg]	4 (6%)
Acute circulatory failure with catecholamine support	54 (75%)
Acute renal failure with renal replacement therapy	11 (15%)

APACHE II: Acute Physiology and Chronic Health Evaluation II, BMI: Body Mass Index; SAPS II: Simplified Acute Physiology Score, NRS: Numeric Rating Scale, CFS: Clinical Frailty Scale; SOFA: Sequential Organ Failure Assessment.

**Table 2:** Energy delivery in the ICU (days 1–7), according to subsequent formulas used to assess the calorie intake.

Day	Formula Used to Assess the Calorie Target	% of BMR Calorie Intake—Median (Q1–Q3)	<70%	70–100%	>100%	n
1	HB	24 (6–44)	69(96%)	3 (4%)	0(0%)	72
	MsJ	24 (6–43)	69(96%)	3 (4%)	0(0%)	
	20 kcal/kg BW	23 (6,47)	69 (96%)	3 (4%)	0 (0%)	
2	HB	64 (33–86)	41 (57%)	18 (25%)	13 (18%)	72
	MsJ	67 (36–91)	38 (53%)	20 (28%)	14 (19%)	
	20 kcal/kg BW	60 (30–90)	44 (61%)	19 (26.5%)	9 (12.5%)	
3	HB	57 (35–93)	43 (62%)	9 (13%)	17 (25%)	69
	MsJ	61 (37–98)	61 (37–98)	61 (37–98)	61 (37–98)	
	20 kcal/kg BW	43 (63%)	43 (63%)	43 (63%)	43 (63%)	
4	HB	72 (34–113)	72 (34–113)	72 (34–113)	72 (34–113)	66
	MsJ	33 (50%)	33 (50%)	33 (50%)	33 (50%)	
	20 kcal/kg BW	16 (24%)	16 (24%)	16 (24%)	16 (24%)	
5	HB	54 (33–102)	54 (33–102)	54 (33–102)	54 (33–102)	60
	MsJ	33 (55%)	33 (55%)	33 (55%)	33 (55%)	
	20 kcal/kg BW	11 (18%)	11 (18%)	11 (18%)	11 (18%)	
6	HB	62 (42–92)	62 (42–92)	62 (42–92)	62 (42–92)	53
	MsJ	30 (57%)	30 (57%)	30 (57%)	30 (57%)	
	20 kcal/kg BW	11 (21%)	11 (21%)	11 (21%)	11 (21%)	
7	HB	69 (48–99)	69 (48–99)	69 (48–99)	69 (48–99)	51
	MsJ	27 (53%)	27 (53%)	27 (53%)	27 (53%)	
	20 kcal/kg BW	14 (27%)	14 (27%)	14 (27%)	14 (27%)	

BH: Harris–Benedict equation; MsJ: Mifflin–St. Jeor equation; BMR: basal metabolic rate, BW: body weight.

**Table 3:** Percent of ESPEN protein target fulfillment (i.e., protein intake 1.3 g/kg BW).

Day	% of Protein Intake—Median (Q1–Q3)	n
Day 1	11 (0–27)	72
Day 2	35 (15–57)	72
Day 3	34 (14–61)	69
Day 4	40 (19–61)	66
Day 5	38 (12,59)	60
Day 6	40 (22–63)	53
Day 7	43 (29–65)	51

**Table 4:** Correlation between acuity of critical illness on ICU admission and delivery of calories and protein on day 4 and day 7.

		Energy–BH	Energy–MsJ	Energy–20 kcal/kg BW	Protein Intake
Day 4	APACHE II	r = −0.08 (*p* > 0.05)	r = −0.07 (*p* > 0.05)	r = −0.06 (*p* > 0.05)	r = −0.07 (*p* > 0.05)
	SAPS II	r = −0.02 (*p* > 0.05)	r = −0.01 (*p* > 0.05)	r = −0.03 (*p* > 0.05)	r = 0.004 (*p* > 0.05)
	SOFA	r = −0.09 (*p* > 0.05)	r = −0.09 (*p* > 0.05)	r = −0.085 (*p* > 0.05)	r = −0.14 (*p* > 0.05)
Day 7	APACHE II	r = 0.14 (*p* > 0.05)	r = 0.14 (*p* > 0.05)	r = −0.11 (*p* > 0.05)	r = 0.21 (*p* > 0.05)
	SAPS II	r = 0.02 (*p* > 0.05)	r = 0.16 (*p* > 0.05)	r = −0.24 (*p* < 0.05)	r = 0.28 (*p* < 0.05)
	SOFA	r = 0.2 (*p* > 0.05)	r = 0.19. (*p* > 0.05)	r = −0.13 (*p* > 0.05)	r = 0.21 (*p* > 0.05)

BH: Harris–Benedict equation; MsJ: Mifflin–St. Jeor equation; APACHE II: Acute Physiology and Chronic Health Evaluation II, SAPS II: Simplified Acute Physiology Score; SOFA: Sequential Organ Failure Assessment, values are Spearman rank coefficients of correlation and ‘*p*’-value (in brackets).

## Data Availability

The data presented in this study are available on request from the corresponding author. The data are not publicly available due to privacy restrictions.
